# A Systematic Review of Parental Occupational Pesticide Exposure and Geographical Proximity to Agricultural Fields in Association with Neural Tube Defects

**DOI:** 10.3390/toxics13010034

**Published:** 2025-01-04

**Authors:** Karoline Felisbino, Shayane da Silva Milhorini, Nathalia Kirsten, Mariane Yoshie Sato, Davi Paula da Silva, Juliana Danna Kulik, Izonete Cristina Guiloski

**Affiliations:** 1 Faculdades Pequeno Príncipe Av Iguaçu, 333, 80230-020 Curitiba, Paraná, Brazil; karoline.felisbino@aluno.fpp.edu.br (K.F.); shayanedasilva.s@gmail.com (S.d.S.M.); nathalia_kirsten123@hotmail.com (N.K.); mariane.sato@aluno.fpp.edu.br (M.Y.S.); davi.silva@aluno.fpp.edu.br (D.P.d.S.); juliana.kulik@hotmail.com (J.D.K.); 2 Instituto de Pesquisa Pelé Pequeno Príncipe, Av Munhoz da Rocha, 490, 80035-000 Curitiba, Paraná, Brazil; 3 Molecular and Cellular Biochemistry, University of Kentucky, Lexington, KY 40356, USA

**Keywords:** malformation, agrochemicals, agricultural fields, farming, pregnancy

## Abstract

Neural tube defects (NTDs) are the second most common congenital anomaly and have been widely associated with exposure to pesticides. This systematic review which analyzes the association between parental exposure to pesticides and NTDs was conducted in accordance with Preferred Reporting Items for Systematic Reviews and Meta-Analysis (PRISMA) guidelines. The search strategy was designed considering the population, exposure, controls, outcomes, and study design (PECOS). The inclusion criteria were epidemiological studies involving pesticides exposure during embryonic development, written in English, Portuguese, or Spanish, and performed in 12 databases. Based on the eligibility criteria, 16 articles were selected for analysis. The most frequently assessed NTDs were anencephaly and spina bifida, with 14 studies investigating each of these conditions. The assessment of pesticide exposure was based on parental occupation and residential proximity to agricultural fields. Studies differed regarding the pesticides assessed, exposure windows, and parents exposed. Regarding the outcomes, it was observed that geographic proximity to agricultural fields and a greater association with family members exposed to pesticides were found in mothers and neonates with NTDs. In relation to occupational exposure, some articles found an association with NTDs, while others did not. Therefore, an analysis of the available evidence suggests that pesticides are a risk factor in the development of NTDs.

## 1. Introduction

Among congenital malformations, neural tube defects (NTDs) are the second most common group of human birth defects [1]. It was estimated that 260,100 pregnancies were affected by NTDs globally in 2015, with an average prevalence of 1.9 cases per 1000 live births [2]. This group of congenital anomalies is divided into open defects, such as anencephaly and myelomeningocele; closed defects, like spina bifida; and herniations, including meningocele and encephalocele [3].

These neural disturbances occur due to a failure in neurulation, which takes place between days 17 and 28 post-fertilization in humans. During this process, the neuropores progressively fuse, completing the neural tube closure. Different types of NTDs correspond to the site where neurulation was disrupted [4]. Most congenital malformations are not linked to a single cause; instead, they arise from the interaction of multiple genetic and environmental factors within a complex etiopathogenesis [5]. Although the pathophysiology of NTDs is not fully understood, several risk factors have been reported: chromosomal syndromes, folic acid insufficiency during the first trimester, maternal diabetes, exposure to valproic acid, and maternal obesity, among others [6].

Furthermore, exposure to organophosphates (OPs), vinclozolin, triazines, and metolachlor has been suggested to increase the risk of teratogenicity [7]. Mothers can be exposed in different ways, including occupational and urban exposure, as well as through food and water [5,7,8]. The most prevalent human congenital abnormalities include musculoskeletal, cardiovascular, and neurological issues, all of which have been associated with NTDs [9].

Our previous review focused on maternal exposure to pesticides during pregnancy and its association with neural tube defects (NTDs), examining factors such as the accumulation in the maternal serum, placenta, and umbilical cord [8]. Although this study offered important observations, it did not address critical aspects, such as fathers’ occupational exposure or proximity to agricultural fields. These gaps underscore the need for more extensive research into these variables, particularly given that individuals living or working near agricultural areas are at higher risk of pesticide exposure. This knowledge gap served as the basis for the present study, which broadens the scope to include both maternal and paternal exposure in occupational and geographical contexts.

Although the effects of pesticides on humans are well documented, there is a notable lack of studies specifically focused on the exposure of pregnant women and their fetuses to these chemical agents [10]. This situation is further compounded by the growing participation of women in agriculture. According to the Food and Agriculture Organization of the United Nations, women constitute 43% of the agricultural workforce in developing countries, with regional variations ranging from 20% in Latin America to 50% in East Asia and Sub-Saharan Africa [11]. In Brazil, women’s participation in the agricultural sector reached 40% [12].

Furthermore, contact with the pesticides can occur indirectly through spraying by tractors and planes over crops, affecting not only agricultural pests but also the soil, surface waters, air, rain, and food, as well as residents of nearby areas [13]. In addition, the contact of a pregnant woman with the father exposed to pesticides can be an indirect form of contamination. Furthermore, the exposure of the father in the periconceptional period can lead to sperm mutations that can lead to malformations. Certain occupations during the periconceptional period can increase the risk of malformations, particularly those involving pesticide exposure [14].

In this context, this study aimed to perform a systematic review of epidemiological research to investigate the relationship between paternal and maternal occupational exposure to pesticides during pregnancy and its association with neural tube defects. Furthermore, this review aims to examine the association between geographic proximity to the agricultural fields and the NTDs.

## 2. Materials and Methods

A systematic literature review was conducted according to the PRISMA guidelines and used the International Prospective Register of Systematic Reviews (PROSPERO) (2024: CRD42024618844). The study protocol was also registered under the Open Science Framework (OSF) database (osf.io/4unq8). The PECOS framework is as follows: Population: newborn children. Exposure: paternal and/or maternal occupational exposure during the periconceptional period or geographical proximity to agricultural fields. Control: no exposure to pesticides during the periconceptional period. Outcomes: neural tube defects. Study design: observational studies.

We performed a literature search from 2000 to 2023 in twelve databases: Science Direct, PubMed, Cochrane Library, Embase, Scopus, Web of Science, BVS/LILACS, EBSCO, ACS Publications, SciELO, Oxford Academic, and Google Scholar. The research question was “Is there an association between maternal and paternal exposure to pesticides during pregnancy (Exposure) and the risk of neural tube defects (NTDs)?” The search query was “pesticides AND (occupational OR exposure) AND neural tube defects”. The exact query for each database is detailed in the Supplementary Materials (Table S1).

Eligible studies must be epidemiological research involving human newborns exposed to pesticides during embryonic development written in English, Portuguese, or Spanish. The exclusion criteria were reviews, editorials, letters, book chapters, conference abstracts, case reports, and studies that did not evaluate the association between occupational pesticide exposure during pregnancy and neural tube defects.

The selection of the articles was made on the Rayyan program. After removing duplicates, two researchers (KF and SSM) independently read the titles and abstracts to select studies that met the eligibility criteria. Disagreement on study inclusion was resolved by a third author (ICG). The following data were extracted by six researchers (KF, SSM, NK, JDK, DPS, and MYS): author, year of publication, country in which the study was carried out, description of the type of study carried out, class of pesticides, agricultural culture, period of exposure, geographic region, and types of neural tube defects

The tools for the quality assessment of the observational cohort and case-control studies from the National Heart, Lung, and Blood Institute (NHLBI) of the National Institute of Health (NIH) (https://www.nhlbi.nih.gov/health-topics/study-quality-assessment-tools, acessed on 15 October 2024.) were used to assess the quality of the selected articles. The evaluation was carried out independently by two investigators: DPS and JDK. Any disagreements were resolved by a third author (ICG). Articles classified as being of good quality (60–100% compliance with the instrument’s items) were considered eligible.

## 3. Results

### 3.1. Studies Included in the Review

**Figure 1:**
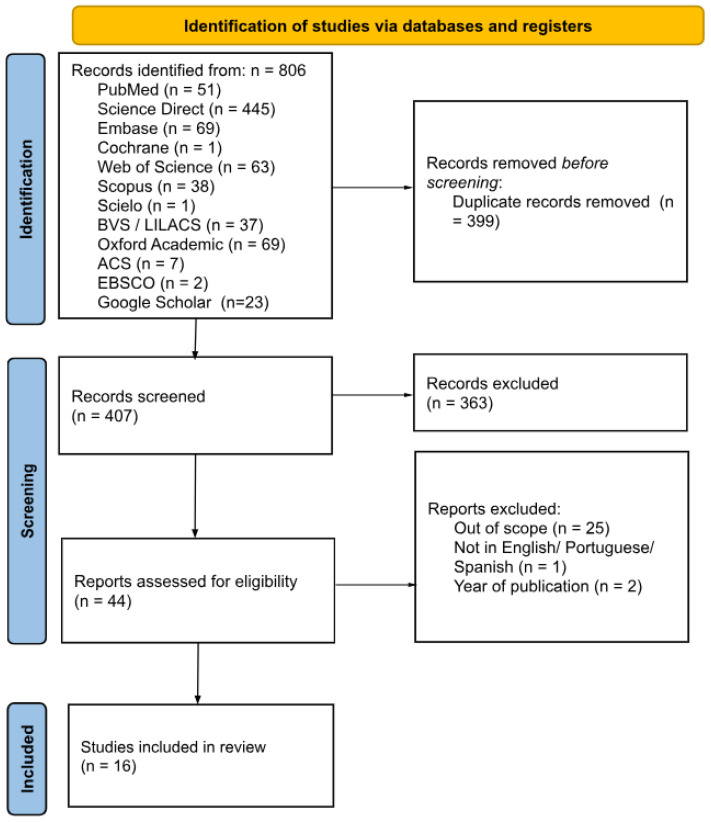
PRISMA 2020 flow diagram for new systematic reviews which included searches of databases and registers only.

The search strategy was presented in a flowchart that illustrates the retrieval process across the different phases of this systematic review (Figure 1). The initial search was conducted in the following databases: PubMed (n = 51), Science Direct (n = 445), Embase (n = 69), Cochrane (n = 1), Web of Science (n = 63), Scopus (n = 38), SciELO (n = 1), BVS-LILACS (n = 37), Oxford Academic (n = 69), ACS (n = 7), EBSCO (n = 2), and Google Scholar (n = 23). This search yielded 806 results, with 407 studies remaining after duplicate removal. Following a review of titles and abstracts for relevance using the Rayyan program, a total of 44 articles were selected. After a full-text review based on eligibility criteria, 28 reports were excluded due to language (n = 1), the wrong publication period (n = 2), or being out of scope (n = 25). The remaining 16 articles were included for analysis.

### 3.2. General

Table 1 summarizes the main characteristics of each study, including the authors, the type of study, alterations (type of NTDs studied), the parent evaluated in the study, the exposure window of the pesticides, and the type of pesticides evaluated in the study.

Most of the studies are from the United States [16,17,21,23,24,26,27,29], two evaluated the Mexican Americans in the U.S. [16,17], and one assessed only pregnancies that occurred in National Priorities List sites in California [23]. There were three Chinese studies [25,28,30]. There was only one study conducted in Norway [22], one in England [18], one in India [19], one in Mexico [20], and one realized in the U.S., with most of its population being American, but there were cases from the United Kingdom, Canada, Pakistan, the Netherlands, and Portugal and controls from the United Kingdom, Canada, Sweden, Mexico, and Australia [15].

Of the 16 studies selected, only 1 was a cohort study [22], 1 was cross-sectional [19], and the remaining 14 were case-control studies. The most evaluated NTDs were anencephaly and spina bifida, with 14 studies assessing each malformation. The following malformation most assessed was encephalocele, with seven studies [16,17,18,19,21,28,30]. Craniorachischisis had three studies [21,26,27] evaluating the malformation, and iniencephaly had two [26,27]. Only one author assessed holoprosencephaly [15], and only one evaluated hydrocephaly [22].

Regarding the exposure to pesticides, ten studies evaluated only maternal exposure [17,19,21,23,25,26,27,28,29,30], one evaluated only paternal exposure [18], and five studies evaluated the exposure of both parents [15,16,20,22,24]. They vary in terms of the period of evaluation of the parents; of the 16 studies, 7 did not inform the window of exposure [18,19,22,23,25,28,30]. Rull and collaborators evaluated the mother through the month before conception and three months after conception [26]. Pettigrew and collaborators used a similar window of exposure: one-month preconception until one-month post-conception for mothers and fathers [24]. Two articles assessed the mothers practically through the same period, but ending two months postconception [21,29]. Another two evaluated the mothers through the period of three months preconception and three months postconception [16,17], but in the evaluation of the paternal exposure, one of the studies assessed the period of six months before to one month after the estimated date of conception [16]. Addissie and collaborators evaluated the mothers and fathers three months before pregnancy and during the entire pregnancy [15]. Lacasaña and collaborators defined two windows of exposure for the mothers and fathers, the acute risk period (ARP), defined as three months before and one month after the last menstruation, and the non-acute risk period (NARP), the exposure occurred before the ARP [20]. Rull and collaborators had two different populations for the study; for the population of 1987–1988, it was defined that the window of exposure was one month before conception and three months after conception, and for the 1989–1991 study population, it was three months before conception and three months after conception [27].

Only two studies reported the specific types of jobs involving pesticide exposure that the participants had [20,21]. Lacasaña and colleagues [20] found a higher number of individuals working as farmers (15 cases vs. 5 controls) and gardeners (5 cases vs. 1 control). In contrast, Makelarski and collaborators [21] observed that most jobs associated with exposure to insecticides and herbicides were in provided services to buildings (janitorial, landscaping, or pest control; 40.7%) or traveler accommodations (26.4%). For those exposed to insecticides + herbicides + fungicides, the most common occupations were in food/drink service places (26.7%), grocery or specialty food stores (18.7%), and agriculture (crop or animal production or support activities for these; 16.7%).

The assessment of the exposure was mostly through the occupation of the parents, and seven studies brought information about the residential proximity to agricultural fields [15,17,22,23,26,27,29]. Five articles assessed the exposure strictly through the occupation of the parent [16,18,20,21,24], three evaluated occupation and other factors of exposure [15,17,22], four assessed the exposure by the quantification of pesticides in the tissue (blood, placenta, and umbilical cord) of the mother and/or the fetus and through a questionnaire that searched for risk behaviors, such as occupation [19,25,28,30]. The studies that evaluated occupational exposure are described in Table 2. Seven articles assessed the exposure of the pesticides through the proximity of the parent’s house to an agricultural field/application of pesticides [15,17,22,23,26,27,29],; the results are shown in Table 3.

The studies also differ in terms of the pesticides evaluated. Four researchers did not specify the pesticide, using a general approach [16,18,20,26], and two evaluated the exposure to insecticides, herbicides, and fungicides isolated and mixed [21,24]. Adissie and collaborators, on the other hand, estimated the exposure through the use of products containing insecticides and herbicides by the mother or anyone in the household; they specified the products, such as personal insect repellants, medications for lice or scabies, pest control products for pets to control for fleas, ticks, and mites, products to control for pests or insects in or outside the home, and herbicides used on the yard/lawn, flowers, vegetables, or fruit trees outside and/or inside the home [15]. Brender and collaborators evaluated the exposure of the mother through the use of insecticides, herbicides, and rodenticides in or outside the home (to kill bugs, flying insects, termites, and rodents or use of bombs and foggers) or on the yard, lawn, or garden (to kill bugs/insects, weeds and rodents) or the self-application of a repellent or anything to kill insects. They also evaluated the exposure of anyone regarding the use in the home, yard, or garden (product self, by the father or professional) [17]. Rull and collaborators evaluated 59 specific restricted-use agricultural pesticides in California (U.S.), including amide, benzimidazole, dicarboximide, dithiocarbamate, halogenated organic, methyl carbamate, organophosphorus, phthalimide, pyrethroid/pyrethrin, substituted benzene, triazine/triazole, and ureas [27]. Nordby and collaborators have the only study that focused on the fungicide Mancozeb [22]. Yang and collaborators assessed 461 individual chemicals and divided them in 62 groups of chemicals, including insecticides, herbicides, and fungicides [29]. Four studies evaluated only the exposure to organochlorines (OCPs) [19,25,28,30]. They vary in evaluating the presence of aldrin, dieldrin, dichlorodiphenyltrichloroethane (DDT), dichlorodiphenyldichloroethylene (DDE), hexachlorocyclohexane (HCH), and endosulfan in the blood of mothers and neonates [19]; an analysis of DDT (and metabolites, DDD, and DDE), Hexachlorocyclohexanes (HCHs), and endosulfan in placenta [25]; an assessment of 25 OCPS in maternal blood: pentachlorobenzene (PeHB), hexachlorobenzene (HCB), HCHs, DDTs, DDEs, DDDs (p,p′-DDD), and heptachlor, aldrin, isodrin, heptachlor epoxide A and B, oxychlordane, α-chlordane, γ-chlordane, endosulfan, dieldrin, endrin, and methoxychlor [28]; and a quantification of 16 OCPs in the umbilical cord, such as heptachlor, aldrin, isodrin, methoxychlor (MOC), HCHs, endosulfan, DDDs, DDEs, and DDTs [30].

### 3.3. Occupational Exposure and NTDs Outcomes

Regarding occupational exposure to pesticides and its possible relationship with the occurrence of NTDs, Addissie and collaborators did not find a relationship between maternal or paternal occupational exposure and the risk of holoprosencephaly, neither for three months before pregnancy or during pregnancy, although the exposure rates observed were low, especially for maternal exposure (Table 2) [15]. However, geographical proximity to agricultural fields resulted in this outcome, which will be addressed in Section 3.4

Brender and collaborators [16] assessed parental occupational exposure using two different methodologies: (1) evaluating occupational histories and (2) self-reporting exposures. The associations between maternal occupational exposure to pesticides and cases of NTD were insignificant, and the methods used to classify exposure had an overall agreement of 98%, with women in the case group being 2.5 times more likely (95% CI, 0.4 to 27.8) to report occupational exposure when compared to those in the control group. With regard to paternal occupational exposure to pesticides, the risk estimates were close to zero for NTDs, and the overall agreement between the two assessment methods used was 92%. However, it is worth noting that the methods differed regarding the OR, with the OR for self-reported paternal exposure being 3.8 (95% CI, 1.2 to 12.6), while the OR obtained when assessing exposure through work history was 1.2. In addition, only 9% of fathers (17% case and 3% control) provided their own occupational information, and the numbers of self-reported paternal occupational exposures to pesticides differed from those identified in the work history assessment.

In another work, Brender and collaborators interviewed case women, whose children were born with some NTDs, and control women, whose children did not have NTDs, five to six weeks after giving birth, asking them about exposure to pesticides [17]. They found no associations between maternal occupational exposure to pesticides and NTDs. However, women in the case group were more likely to live with family members who had a higher chance of occupational exposure to pesticides (OR 3.1; 95% CI, 1.4–6.6).

On the other hand, Fear and collaborators observed a significant association between paternal occupational exposure to pesticides and the risk of NTDs, and this group included farmers (15 cases and 5 controls) and gardeners (5 cases and 1 control). However, the authors emphasize that the intake of an adequate level of folate by pregnant women reduces the risk of NTDs, but it is not known whether the partners of the men participating in the study reached these levels [18].

Kalra and collaborators evaluated the presence of certain organochlorine pesticide residues in the blood of mothers and neonates, namely, aldrin, dieldrin, dichlorodiphenyltrichloroethane (DDT), dichlorodiphenyldichloroethylene (DDE), hexachlorocyclohexane (HCH), and endosulfan [19]. They observed that exposure to some pesticides (endosulfan, DDT, and DDE) may be associated with cases of NTDs. The authors found that mothers who gave birth to newborns with NTDs had significantly higher levels of t-HCH, DDE, and endosulfan compared to mothers in the control group, while neonates with NTDs not only had significantly higher levels of t-HCH, DDE, and endosulfan but also DDT, compared to neonates without NTDs. Curiously, despite the correlation between levels of certain pesticides and cases of NTDs, there was no occupational difference between cases and controls.

In order to investigate if occupational exposure to pesticides was related to cases of NTDs, Lacasaña and collaborators investigated, through questionnaires for parents and access to the Epidemiological Surveillance System of Neural Tube Defects (ESSNTD) in Mexico, whether maternal or paternal exposure to pesticides would lead to a higher risk of having a child with anencephaly [20]. To this end, the authors evaluated the exposure in different periods: the acute risk period (ARP), which was defined by three months before and one month after the last menstrual periods; and the non-acute risk period (NARP), which was the exposure prior to the above-mentioned period. The authors found that maternal exposure during ARP generated a higher risk of having a child with anencephaly, with an adjusted odds ratio (aOR) = 4.58 and a confidence interval (CI) of 1.05 to 19.96. On the other hand, women who worked in agriculture during NARP showed a much lower increase in risk (aOR = 1.65 [95% CI 0.43 to 6.39]). This emphasizes that the period during which a woman is exposed to pesticides is a relevant factor for the outcome to be observed. Paternal exposure showed that the intensity of exposure is relevant to the outcome, since fathers who apply pesticides, who are exposed more intensely, had a higher risk of conceiving a child with anencephaly, although this was not significant, when compared to exposure in agricultural workers who did not apply pesticides. In addition, the exposure of both parents to agricultural work during the NARP period was associated with NTDs (crude OR = 4.82 [95% CI 1.00 to 23.29]); however, the values were not adjusted for confounding factors due to the low sample size of this group.

On the other hand, Makelarski and collaborators investigated whether maternal occupational exposure to pesticides during the periconceptional period and the occurrence of neural tube defects in the child were related. They obtained a positive aOR, although not significant or marginally significant, for exposure to insecticides + herbicides for all cases of NTD combined, in addition to spina bifida alone. The positive association was also noted for any exposure and cumulative exposure to insecticides + herbicides + fungicides, both for anencephaly alone and for encephalocele alone. The authors also assessed maternal cumulative exposure (0, >0 to <50%, ≥50%) to any pesticide or to insecticides alone and obtained aORs close to unity for both the combined NTD cases and the subgroups. Thus, although only a statistically imprecise increase was obtained, the researchers concluded that the associations between exposure and cases of NTD are dependent on the class of pesticides, in addition to varying according to the NTD subtype [21].

Nordby and collaborators investigated whether the application of mancozeb, which normally occurs on potato farms, influenced the risk of NTDs [22]. The authors found that although no significant influence on maternal exposure and the occurrence of NTDs was found when the father worked on the farm for more than 500 h a year, there was a higher risk of the child being born with an NTD. In addition, potato cultivation was positively associated with the occurrence of anencephaly (PRR=1.66 [95%CI=0.88–3.12]) and hydrocephalus (PRR = 1.98 [95%CI = 1.00–3.93]).

Differently, Pettigrew and collaborators assessed whether paternal, maternal, or both parents’ occupational exposure to pesticides would increase the chance of a child being born with spina bifida [24]. The authors found a positive association when fathers were exposed to a combination of insecticides and pesticides. However, almost all other aORs for paternal exposure (insecticides only, fungicides only, herbicides only, insecticide + herbicide, fungicide + herbicide or insecticide + herbicide + fungicide) were close to unity. Interestingly, although paternal (aOR = 0.8 [95% CI 0.6–1.8]) or maternal (aOR = 0.8 [95% CI 0.5–1.3]) exposure to any pesticide did not lead to an increased risk of spina bifida, this correlation was found when both parents were exposed to pesticides (aOR = 1.5 [95% CI 0.9–2.4]).

In addition to assessing exposure to pesticides through questionnaires and blood analysis, the placenta can also be used for this purpose. In this context, Ren and collaborators investigated the possible association between persistent organic pollutants, including pesticides, and the risk of NTDs by measuring their concentration in the placenta [25]. They found that the concentrations of o, p′-DDT and its metabolites (o, p′-DDE and o, p′-DDD, or the sum Σ3o, p′-DDTs), α-HCH, γ-HCH, and endosulfan were significantly higher in the placentas of cases compared to those of controls. In addition, it was noted that the isomerism of the substance could also influence accumulation, since although α-HCH and γ-HCH were more concentrated in the placenta of the cases, this difference was not found for β-HCH. In addition, the cases showed a lower ratio of the metabolites (p, p′-DDE + p, p′-DDD) to the parent (p, p′-DDT) compared to the control group. Higher levels of γ-HCH resulted in a 3.36-fold increase (95% CI, 1.10–6.44) in the risk of any NTD occurrence, which changed to 11.75 after adjusting the data for confounding factors (95% CI, 2.95–46.88), while for α-endosulfan, the increase was 2.53-fold (95% CI, 1.03–5.99), 3.26 after adjustment (95% CI1.10–9.71). Interestingly, despite these differences, it was found that the mother’s occupation on farms did not increase the risk of NTDs.

Wang and collaborators evaluated whether maternal serum OCP levels were related to an increased risk of NTDs in their offspring. The authors found that although the overall serum OCP concentration of mothers of the cases was higher than that found in mothers of the controls offsprings, no dose–response relationship between OCPs and NTD risk was observed. Therefore, clear associations between maternal serum OCPs and NTDs occurrence were not observed in the study population. In addition to total serum concentrations of OCPs, HCB levels were also higher for cases—more specifically, for anencephaly, while for p, p′-DDE and α-HCH, no difference was found. Additionally, maternal occupation on farms was not associated with NTD outcomes [28].

Yin and collaborators evaluated whether prenatal exposure to OCPs could be associated with the development of NTDs [30]. It was obtained that exposure to β-hexachlorocyclohexane increased the risk of NTDs by 5.44-fold (95% CI, 2.21–13.41), endosulfan I increased it by 2.51-fold (95% CI, 1.07–5.86), endosulfan II increased it by 3.78-fold (95% CI, 1.60–8.89), ρ, ρ’-dichlorodiphenyldichloroethane increased it by 3.42-fold (95% CI, 1.44–8.12), and ρ, ρ’-dichlorodiphenyltrichloroethane increased it by 2.89-fold (95% CI, 1.22–6.8w6). Furthermore, a suggestion of joint effects of OCPs was found, since the risk of NTDs increased with the concentrations of the 16 OCPs evaluated as a mixture. However, although these correlations were observed, no association was obtained between occupational exposure and NTD cases.

The variable used to adjust the data providing the adjusted odds ratio (aOR) changed depending on the article. Most of them included maternal age [15,16,18,20], passive or active smoking [15,16], use of tobacco [20], alcohol use [15,20], illicit drugs use [20], folic acid use [15,20], maternal education [16,20,24], race/ethnicity [24], marital status, family income [20], body mass index [16], multiplicity of birth [18], number of previous obstetric events [18,20], history of illness, history of stillbirths, spontaneous abortions, premature births, and malformed children, antenatal care in the index pregnancy, family reproductive history [20], time from estimated date of survey delivery [15], year of index pregnancy, and gender of baby [18].

The types of occupational exposure assessed in the articles were farmers, farm managers, gardeners, and groundsmen [18]; agricultural or agrarian [19]; agricultural work in general [20]; farmers only [28,30], agricultural work, maintenance workers, and property managers [24]; food/drink service places, grocery or food stores, and agriculture [21,22]; or only reported occupational exposure, without specifying the occupation [15,16,17,25].

### 3.4. Geographical Proximity to Agricultural Fields

Ten articles [16,18,19,20,21,22,24,25,28,30] did not provide information regarding geographical proximity to agricultural fields. In contrast, six articles [15,17,23,26,27,29] present different information on the association between parents living in regions close to agricultural fields (Table 3).

Addissie and collaborators revealed a significant association between living within 100 m of agricultural fields and an increased risk of NTDs, with an aOR of 3.24 (CI 0.94–12.31) [15]. Similarly, Brender and collaborators [17] found significant associations with proximity to agricultural fields and the frequency of walking through those fields. Living less than 400 m from fields was associated with an OR of 3.6 (CI 1.7–7.6) for NTDs and walking through fields had an OR of 4.7 (CI 1.0–30.2).

The study by Orr et al. [23] also found significant associations, particularly with pesticide exposure. They reported an OR of 3.06 (95% CI: 1.39–6.71) for anencephaly and an OR of 1.47 (95% CI: 0.69–3.12) for all NTDs. Similarly, the study by Rull and collaborators [26] showed that exposure to vineyards, measured through land-use surveys, was particularly associated with an increased risk of NTDs, with significant odds ratios at 400 m (OR = 2.45; CI = 1.08–5.58) and 500 m (OR = 2.04; CI = 1.01–4.13). Additionally, significant associations were found for proximity to nonpermanent crops (OR = 1.62; CI = 1.08–2.43) and orchards (OR = 1.95; CI = 1.14–3.34) when self-reported at 400 m.

Another study by Rull and collaborators [27] found that pesticide exposure within 1000 m of agricultural fields was associated with an increased OR of neural tube defects, with various pesticides showing significant associations, such as an OR of 3.3 for spina bifida with amides and an OR of 2.7 with benzimidazoles. On the other hand, the study by Yang and collaborators [29] indicated that proximity to areas of pesticide application during pregnancy may be associated with a significant increase in the risk of birth defects, particularly spina bifida, with an OR of 5.1 (CI 1.7–15.6). Furthermore, the authors found that exposure to the herbicide bromoxynil (hydroxybenzonitrile) was higher among cases of spina bifida (5.69%) compared to controls (1.3%).

Regarding the cultivars planted, three authors did not inform on the type of crop [15,17,23], while two reported that they were non-permanent crops, orchard crops, and vineyards [26,27]. Yan et al. [29], on the other hand, evaluated infrequently rotated crops such as orchards and vineyards and frequently rotated crops.

### 3.5. Quality Assessment

We conducted the results quality assessment of the risk of bias for the studies according to the National Heart, Lung, and Blood Institute (NHLBI) tool shown in Table 4 (for the case control studies) and Table 5 (for the observational cohort study). The estimated percentage of all the included studies was in the range of 70% to 100%, and all 16 studies were rated as being of good quality. Details of the risk of bias assessment are available in Tables S2 and S3.

## 4. Discussion

### 4.1. General

According to the World Health Organization, more than 300,000 newborns are born with neural tube defects (NTDs) each year in the world. The most common NTDs are anencephaly, spina bifida, and encephalocele [31]. Accordingly, the most evaluated NTDs were anencephaly and spina bifida, with 14 out of 16 studies assessing each malformation. Craniorachischisis and iniencephaly are relatively rare malformations [6]. The cause and pattern of malformations are related to the environment of the mother, including the geographic location, genetic interactions, and maternal health/nutrition status [32]. The prevalence of NTDs varies regionally and is higher in developing countries, with countries in Africa and Asia reporting the highest rates, being responsible for 85% of all NTD-associated stillbirths globally [6]. In low-income and middle-income countries, the impact caused by NTDs is greater than in developed countries, and even with programs and guidelines for prevention and diagnoses in early pregnancy, it is still a challenge for the health system [33]. But only 4 studies, out of 16 reported in this review, are from developing regions [19,25,28,30]. Surveillance systems are crucial to gathering prevalence data, identifying risk factors, promoting research development in the field, creating prevention strategies, and planning for services and referrals for affected children to medical, educational, and social services [34], but it is still a challenge for developing countries to develop great working surveillance systems, which are crucial to understanding and preventing NTDs [6].

In this review, most of the studies are from the U.S., a country that is considered developed and high-income [35]. The U.S. does not have a surveillance system for birth defects, but most states have it [36]. The study by Zangajor and collaborators [35] claimed that the prevalence rate of NTDs (anencephaly, spina bifida, and encephalocele) is 5.2 per 10,000 births, considered low. More recently, Stallings et al. [36] showed a prevalence of 1.91 for anencephaly, 0.95 for spina bifida, and 3.48 for encephalocele (per 10,000 live births). It is important to note that most of the studies are from countries that have a low prevalence of NTDs, whilst the countries with more concerning numbers of these malformations are the least studied in comprehending the development and causes of these diseases, such as the exposure of pesticides, considered in this review. For example, this review includes no studies from Latin America, including large countries, such as Brazil, highlighting the limited attention given to this issue in developing countries.

The closing of the neural tube occurs during the 17th and 28th days post-fertilization of the embryo. However, some processes happen early, which can influence the correct development of the neural tube and, posteriorly, the formation of the neural system. Many factors can interfere with this process, such as genetic factors and exposure to contaminants, such as pesticides [4,37]. The studies included in this review analyzed different windows of exposure to pesticides, and some did not inform, which can be related to associations or no associations between NTDs and pesticides. The complexity of the epidemiology of these malformations may cause confusion when choosing the period of the evaluation of the parents. In addition, seven studies [18,19,22,23,25,28,30], despite having reported maternal or paternal exposure to pesticides, did not detail the period of exposure. The window of exposure plays a crucial role in understanding malformations, and the non-evaluation of these variables can lead to misleading information and conclusions, representing a significant limitation of this review.

### 4.2. Occupational Exposure and NTDs Outcomes

Among 16 articles covered in this study, 12 evaluated the relationship between NTDs and occupational exposure to pesticides. Numerous scientific studies have linked such exposure among agricultural workers to various dysfunctions and diseases, such as genetic damage [38], an increased risk of dementia and Alzheimer’s disease in late life [39], diabetes [40], cancer [41], negative changes in neurobehavioral health [42], respiratory health [43,44], and kidney function [45].

In addition to the occupational exposure and health risks for farmworkers mentioned above, it is crucial to consider instances when pregnant women work directly on farms and are exposed to pesticides or where paternal exposure occurs when fathers work on farms. In such cases, apart from the risks to maternal and paternal health, there are also concerns regarding the proper development and formation of the fetus. Some studies indicate a possible relationship between exposure to pesticides during pregnancy and the development of musculoskeletal, urogenital, and cardiovascular abnormalities, in addition to neural tube defects [9]. In a previous study, maternal occupational exposure was linked to the development of NTDs [8]. However, paternal exposure was not considered in that investigation. Herein, we assess how both maternal and paternal exposure, or exposure from both parents concomitantly, may be associated with cases of NTDs, either through occupational exposure to pesticides or living near agriculture fields.

Among the 12 epidemiological studies covered in this study investigating the correlation between occupation and cases of NTDs, two indicated an association between maternal occupational exposure and the outcome of NTDs [20,21]. It is worth noting that both studies highlighted the importance of evaluating the exposure in a more specific and categorized way. Lacasanã and collaborators observed that exposure during the ARP period, corresponding to three months before and one month after the last menstruation period, resulted in a higher risk of developing NTDs, emphasizing a greater sensitivity during this period [20]. On the other hand, Makelarski and collaborators demonstrated that different types of pesticides have a different influence on the risk of NTDs. When exposure to pesticides in general was analyzed, no correlation with NTDs was found. However, when they investigated exposure to specific combinations of different groups of pesticides, namely, insecticide + herbicide or insecticide + herbicide + fungicide, a correlation was found, suggesting a complementary effect of these mixtures [21]. Furthermore, in addition to the categorization by type of pesticide, the researchers observed that some subgroups of NTDs were more likely to develop depending on the type of pesticide the mother was exposed to. For example, if exposed to insecticide + herbicide, a higher risk of spina bifida was observed, while exposure to insecticide + herbicide + fungicide led to the risk of anencephaly or encephalocele.

On the other hand, three studies have indicated an association between paternal occupational exposure to pesticides and NTDs [18,20,22]. The work developed by Lacasanã and collaborators [20] indicated that the level of exposure is an important factor in the outcome of NTDs, since for regular farmworkers, no association was found, either during the ARP or NARP period; however, when the father worked as an applicator, being exposed to a greater quantity of pesticides, a positive association, although not significant, was found with NTDs occurrence. The studies by Fear et al. [18] and Nordby et al. [22], on the other hand, did not specify periods of gestation when exposure occurred but only considered the profession of the participants.

Aside from the isolated occupational exposure of the mother or father, both parents may also be exposed to agricultural work, as reported in two articles [20,24]. In both cases, the exposure of both parents was associated with the risk of NTDs. Interestingly, in the work by Pettigrew and collaborators [24], isolated maternal or paternal exposure to any pesticide was not associated with NTDs, which changed when both parents were exposed, emphasizing the importance of assessing different exposure scenarios for a proper and more realistic evaluation of occupational risks to the fetus.

On the other hand, seven articles found no difference between cases and controls regarding maternal and/or paternal occupation [15,16,17,19,25,28,30]. However, it is important to emphasize that, among these, four articles realized chemical analyses to measure pesticide concentrations, whether through the blood [19], serum [28], placental tissue [25], or umbilical cord [30]. In all of these articles, correlations were found between higher concentrations of specific types of pesticides, such as endosulfan, DDT, and DDE [19,25], and OCPs [28,30] in the mothers of cases compared to mothers in the control group and DTNs cases. This indicates that although occupation was not a determining factor in these works, exposure to pesticides is still associated with DTNs outcomes. Additionally, it is worth noting that for all seven articles, whether those with chemical analysis or those without, occupational exposure was accessed via questionnaires or interviews, which may have influenced memory bias in self-reporting. It is also worth noting that although Addissie et al. [15] and Brender et al. [17] did not find a relationship between occupational exposure and NTDs, this association was observed for geographical proximity to agricultural fields (Table 3). However, the other five studies [16,19,25,28,30] did not investigate whether there was a statistical difference in relation to the geographical proximity of cases and controls to agricultural fields, which could justify the higher level of pesticides found in cases when compared to controls, except Brender et al. [17], who did not evaluate the level of pesticides. In addition, in the articles in which chemical analyses were carried out [19,25,28,30], although the number of mothers working on farms was similar in both the case and control groups, the authors did not clarify whether the higher pesticide levels in the cases were linked to mothers who worked on farms. Therefore, despite the equal number of farm working in both groups, it cannot be concluded that their exposure levels were comparable.

### 4.3. Geographical Proximity to Agricultural Fields

Spatial indicators, such as proximity to fields, area of cultivation, and pesticide application in the surrounding areas, are often associated with increased exposure to these products. This exposure primarily occurs due to the impact of drift transport, influenced by meteorological, topographical, and behavioral variables [46]. Thus, even if an individual does not work directly in agriculture, they can be highly exposed to pesticides simply by living or frequently moving near these areas.

Although few of the reviewed articles have investigated the association between NTDs and the geographical proximity of cultivation areas, all studies reported a significant association [15,17,23,26,27,29]. The distances assessed varied considerably among the studies, highlighting an important finding: even at greater distances, such as 1000 m, analyzed by Rull and collaborators [27], a significant association with NTDs was identified, just as in the study by Addissie and collaborators [15], which examined a much shorter distance of only 100 m.

Another important issue, addressed only by Rull et al. [26], concerns the type of crop near the exposed pregnant women. They demonstrated that residing near crops such as non-permanent crops, orchards, and vineyards is significantly related to an increased risk of developing NTDs. Among the three types of crops analyzed by the authors, vineyards presented the highest risk, which can be explained by the high sensitivity of grapes to pests; the quality of the fruit is directly linked to the health of the plants [26]. To ensure this quality, a wide variety and quantity of pesticides are used [47]. Additionally, the concentrations applied are often high, frequently exceeding permissible limits [48]. Compared to other crops, such as wheat in France, vineyards have a higher average treatment frequency index (TFI) [49].

In Brazil, one of the largest users of pesticides in the world, vineyards also require a higher quantity of pesticides compared to other crops, such as oranges, coffee, and bananas [50]. However, besides the concern with vineyards, it is crucial to consider the risks associated with other crops, such as tomatoes and bell peppers, which exhibit the highest concentrations of pesticides in Brazil [51] Although Brazil is often mentioned for its high pesticide use, to date, no studies have assessed exposure to these pesticides and the risk of NTDs. Therefore, new research must be conducted to investigate this relationship and ensure public health safety in more regions beyond those observed in the studies found in this systematic review.

Furthermore, Rull and collaborators [26] worked with self-reports and land-use surveys. Although self-reporting showed greater significance than land-use surveys, both demonstrated an association. The study highlights that both self-reporting and land-use maps have limitations; self-reports can be inaccurate due to memory failure or subjective perception, and the maps may not capture temporal and spatial changes in crops [52]. Therefore, in areas with frequent crop rotation, such as non-permanent ones, the combination of these methodologies, while imperfect, can provide a more comprehensive overview of pesticide exposure, especially in regions where geospatial data are not frequently updated. More studies utilizing both technologies should be conducted in different regions.

Another issue addressed by Rull et al. [27] was the comparison of risk among different types of pesticides. The study highlighted that the type of pesticide, in addition to the amount, mixture, and frequency of application, are determining factors that can increase or reduce the risk of congenital defects. For example, it was observed that the risk of anencephaly is higher with exposure to organophosphates (OR = 1.6, CI: 1.0–2.5), while the highest risk of spina bifida is associated with amides (OR = 3.3, CI: 1.2–9.3), suggesting that the impact of exposure may vary depending on the pesticide.

Yang et al. [29] pointed to a significant increase in the risk of spina bifida related to exposure to the pesticide Bromoxynil (hydroxybenzonitrile). Additionally, while the associations with anencephaly did not reach statistical significance, some results suggest a trend worth considering. The chemical group avermectin showed an OR of 2.5, with a CI of 1.0 to 6.4, while neonicotinoids had a similar OR of 2.5, with a CI ranging from 0.9 to 7.1. The group of salts or esters of dichlorophenoxyacetic acid (2,4-D and dichlorprop) showed an OR of 2.0, with the CI varying from 0.8 to 5.1. Meanwhile, the polyalkylene oxide compounds presented an OR of 1.7, with a CI between 0.9 and 3.0. These results, while not statistically significant, indicate a possible trend of risk that deserves further exploration [29].

Moreover, Brender and collaborators emphasize that, in addition to living near agricultural areas, walking through fields can significantly increase the risk of NTDs. Thus, a possible prevention strategy would be to advise pregnant women, especially during the preconception period, to avoid these areas exposed to pesticides [17]. Finally, the study by Orr et al. compared risks by separating groups by ethnicity, suggesting an association between the exposure of pregnant women to hazardous waste in contaminated areas and an increased risk of congenital defects, especially NTDs, among racial/ethnic minorities [23]. This reinforces the importance of further investigating the relationship between environmental factors and NTDs in different populations and contexts.

Additionally, each reviewed study adopted different contexts for comparison: while Rull et al. [26] focused on the types of crops and pesticides near pregnant women, others, such as Yang et al. [29], analyzed the risk of NTDs based on specific types of pesticides, and Orr et al. [23] separated the comparison groups by ethnicity, investigating the impact of exposure to hazardous waste in racial minorities. These diverse approaches underscore the need to consider multiple variables (regional, environmental, and demographic) when evaluating the risks of NTDs.

It was noted that studies examining exposure based on proximity to agricultural fields showed greater consistency with each other compared to those assessing occupational exposure. This underscores the importance of considering participants’ addresses in epidemiological studies investigating potential associations between NTDs and environmental exposures. Furthermore, the limited number of studies—only six—highlight the need for additional research to better understand this association.

Ultimately, with regard to the limiting factors of studies that have addressed exposure due to geographical proximity, we would highlight self-reporting bias [15,17,27], the possible change in the residence of mothers during the course of the study [23], and the limitations of land-use surveys and geocoded address data, including changes in land use [26].

## 5. Conclusions

It can be considered that there are indications that pesticide exposure is associated with NTDs cases, since 13 of the 16 articles covered by this study found this association. Regarding occupational exposure, five articles found a link with NTDs, while seven did not find this correlation. However, among the seven that did not find an association, five showed that the concentration of pesticides in the mother in the cases group was higher when compared to the mothers in the control group, which indicates that, although there was no difference in occupation between the control and case groups, the mothers who gave birth to newborns with NTDs were more exposed to pesticides. This fact may be due to a geographical proximity to agricultural fields, which was not explored in these specific studies. Therefore, the need to consider both geographical and occupational exposure in the same research is highlighted to avoid bias. From the 16 articles used in this study, only 6 investigated the association between geographical proximity to agricultural fields and NTDs, and all of them found a correlation. Additionally, despite it being known that NTDs are more recurrent in less developed areas, most of the studies were carried out in developed countries, mainly in the United States, which highlights the need to investigate the association between NTDs and pesticide exposure in developing and underdeveloped countries as well.

## Figures and Tables

**Table 1:** General characteristics of the studies included in the review.

Type of Studies	Country	NTDs	Cases/Controls	Parent Evaluated	Exposure Period	Type of Exposure	Pesticides Evaluated	Reference
Case control	USA, UK, Canada, Pakistan, Netherlands, Portugal, Sweden, Mexico, Australia	Holoprosencephaly	92 cases; 56 controls.	Mother and father	3 months before pregnancy and first, second, and third trimesters of pregnancy.	Use of products containing pesticide by the mother or by anyone else in the household; Proximity to an agricultural field; Occupational	Insecticides; Herbicides.	[15]
Case control	USA	Anencephaly, spina bifida, and encephalocele	184 cases; 225 controls.	Mother and father	Mother: 3 months before and 3 months after conception; Father: Occupational exposures 6 months before to 1 month after the estimated date of conception.	Occupational	NI	[16]
Case control	USA	Anencephaly, spina bifida, and encephalocele	249 cases; 498 controls.	Mother	3 months before and 3 months after conception;	Home use, self-application of repellent, yard or garden use; Proximity to agricultural fields; Occupational	Insecticides; Herbicides; Rodenticides.	[17]
Case control	England	Anencephaly, encephalocele, spina bifida aperta or spina bifida oculta	694 cases; 594 controls.	Father	NI	Occupational	NI	[18]
Cross-sectional	India	Anencephaly, encephalocele, open (spina bifida aperta associated with Chiari malformation and ventriculomegaly, myelomeningocele, and myeloschisi) or closed (lipomyelomeningocele, meningocele, myelocysto- cele, spina bifida occulta, dermal sinus, and neuroenteric cyst)	35 cases; 35 controls.	Mother	NI	NI	OCPs—Aldrin, Dieldrin, Endolsulfan, DDT, DDE, HCH	[19]
Case control	Mexico	Anencephaly	157 cases; 151 controls (mothers). 129 cases; 110 controls (fathers).	Mother and father	Acute risk period (ARP): three months before and one month after the last menstruation; Non-acute risk period (NARP): exposure occurred before the ARP.	Occupational	NI	[20]
Case control	USA	Anencephaly, craniorachischisis, spina bifida, or encephalocele	502 cases; 2950 controls.	Mother	1-month preconception through 2 months postconception	Occupational	Insecticides, herbicides, and fungicides	[21]
Cohort	Norway	Anencephaly, spina bifida, and hydrocephaly	131 cases; 102,703 controls.	Mother and father	NI	Proximity to the agricultural field; Occupational	Mancozeb (fungicide)	[22]
Case control	USA	CNS groupings NTDs, anencephaly, and Spina bifida	359 cases; 14,463 controls.	Mother	NI	Housendry location	Pesticides, including DDT (1,1,1-trichloro-2,2-bis(p-chlorophenyl) ethane) types, cyclodienes, other chlorinated pesticides, organophosphates, and carbamates.	[23]
Case control	USA	Spina bifida	469 cases; 2745 controls.	Mother and father	1-month preconception through 1 month postconception	Occupational	Herbicides, insecticides, and fungicides	[24]
Case control	China	Anencephaly and spina bifida	80 cases; 50 controls.	Mother	NI	NI	OCPs—DDT, HCH, endosulfan	[25]
Case control	USA	Anencephaly, spina bifida cystica, craniorrhachischisis, and iniencephaly	227 cases; 424 controls.	Mother	During the period between the month before conception and 3 months after conception.	Residential proximity to crops	NI	[26]
Case control	USA	Anencephaly, spina bifida cystica, craniorrhachischisis, and iniencephay.	731 cases; 940 controls.	Mother	1987–1988 study population: 1 month before conception and 3 months after conception; 1989–1991 study population: 3 months before conception and 3 months after conception.	Residential proximity to crops	59 insecticides and fungicides of restricted use	[27]
Case control	China	Anencephaly, spina bifida, and encephalocele	117 cases; 121 controls.	Mother	NI	NI	25 OCPs	[28]
Case control	USA	Anencephaly and spina bifida	763 cases; 785 controls.	Mother	1 month preconception through 2 months postconception	Residential proximity to agricultural pesticide applications	Insecticides, herbicides, and fungicides	[29]
Case control	China	Anencephaly, spina bifida, and encephalocele	119 cases; 119 controls.	Mother	NI	NI	16 OCPs	[30]

NI: Not informed.

**Table 2:** Relationship between maternal or paternal occupational exposure to pesticides and the occurrence of NTDs in offspring.

Maternal Exposure n (%)	Maternal Exposure aOR, OR or PPR ^a^ (95% CI)	Paternal Exposure n (%)	Paternal Exposure aOR, OR or PPR ^a^ (95% CI)	NTDs	Outcome	Reference
3 months before pregnancy: Exposed: Cases: 8 (13.1) Controls: 2 (5.7) Non-exposed: Cases: 53 (86.9) Controls: 33 (94.3) During pregnancy: Exposed: Cases: 6 (10) Controls: 3 (8.8) Non-exposed: Cases: 54 (90) Controls: 31 (91.2)	aOR: 3 months before pregnancy: 1.95 (0.07–52.19) During pregnancy: 1.15 (0.11–11.42)	3 months before pregnancy and/or during pregnancy: Exposed: Cases: 16 (27.1) Controls: 5 (14.3) Non-exposed: Cases: 43 (72.9) Controls: 30 (85.7)	aOR: 3 months before pregnancy and/or during pregnancy: 2.39 (0.34–20.58)	Holoprosencephaly	No association was found between occupational exposure and the risk of holoprosencephaly	[15]
Exposed: ^d^ Cases: 4 (2.2) Controls: 4 (1.8)	aOR: 1.2 (0.3–4.8)	Exposed: ^d^ Cases: 12 (7.5) Controls: 13 (6.3)	aOR: 1.2 (0.5–2.8)	Anencephaly, spina bifida, and encephalocele	No association was found between maternal and paternal occupational exposure and NTDs.	[16]
Exposed: ^d^ Cases: 6 (3.3) Controls: 5 (2.2)	aOR: 1.9 (0.50–7.1)	NI	NI	Anencephaly, spina bifida, and encephalocele	No association was found between maternal occupational exposure and NTDs.	[17]
NI ^b^	NI	Exposed: ^d^ Cases: 20 (3) Controls: 7 (1)	aOR: 2.69 (1.09–6.65)	Anencephaly, encephalocele, spina bifida aperta, or spina bifida occulta	Exposure to pesticides led to a significantly elevated association with the risk of NTDs.	[18]
Exposed: ^d^ Cases: 8 (22.8) Controls: 8 (22.8)	OR: 1 (0.33–3.05)	Exposed: ^d^ Cases: 12 (34.9) Controls: 15 (42.8)	OR: 0.70 (0.26–1.83)	Anencephaly, encephalocele, open (spina bifida aperta associated with Chiari malformation and ventriculomegaly, myelomeningocele and myeloschisi), or closed (lipomyelomeningocele, meningocele, myelocysto- cele, spina bifida occulta, dermal sinus, and neuroenteric cyst)	Exposure to pesticides (endosulfan, DDT, and DDE) may be associated with cases of NTDs. But no difference related to occupation was found between cases and controls.	[19]
Cases: ^d^ Exposed some moment in life: Non-agricultural workers: 110 (72.85) Agricultural workers: (41) 27.15 Exposed during acute risk period (ARP): Non-agricultural workers: 110 (89.43) Agricultural workers: 13 (10.57) Exposed during non-acute risk period (NARP): Non-agricultural workers: 110 (80.29) Agricultural workers: 27 (19.71) Controls: ^d^ Exposed some moment in life: Non-agricultural workers: 125 (82.78) Agricultural workers: 26 (17.22) Exposed during acute risk period (ARP): Non-agricultural workers: 125 (93.98) Agricultural workers: 08 (6.02) Exposed during non-acute risk period (NARP): Non-agricultural workers: 125 (88.03) Agricultural workers: 17 (11.97)	aOR: Exposed some moment in life: Non-agricultural workers: 1 Agricultural workers: 1.47 (0.79–4.93) Exposed during acute risk period (ARP): Non-agricultural workers: 1 Agricultural workers: 4.57 (1.05–19.96) Exposed during non-acute risk period (NARP): Non-agricultural workers: 1 agricultural workers: 1.65 (0.43–6.39)	Cases: ^d^ Exposed some moment in life: Non-agricultural workers: 48.06 (62) Agricultural workers: 26.36 (34) Applicators: 25.58 (33) Exposed during acute risk period (ARP): Non-agricultural workers: 64.58 (62) Agricultural workers: 20.93 (20) Applicators: 14.58 (14) Exposed during non-acute risk period (NARP): Non-agricultural workers: 65.26 (62) Agricultural workers: 14.74 (14) Applicators: 20 (19) Controls: ^d^ Exposed some moment in life: Non-agricultural workers: 67 (60.91) Agricultural workers: 29 (26.36) Applicators: 14 (12.73) Exposed during acute risk period (ARP): Non-agricultural workers: 67 (75.28) Agricultural workers: 13 (14.61) Applicators: 9 (10.11) Exposed during non-acute risk period (NARP): Non-agricultural workers: 67 (76.14) agricultural workers: 16 (18.18) Applicators: 5 (5.68)	aOR: Exposed some moment in life: Non-agricultural workers: 1 Agricultural workers: 0.66 (0.30–1.48) Applicators: 2.17 (0.86–5.49) Exposed during acute risk period (ARP): Non-agricultural workers: 1 Agricultural workers: 0.79 (0.257–2.36) Applicators: 2.5 (0.73–8.64) Exposed during non-acute risk period (NARP): Non-agricultural workers: 1 agricultural workers: 0.61 (0.21–1.74) Applicators: 2.03 (0.58–7.08)	Anencephaly	Mothers who worked in agriculture during the ARP had a higher risk of having children with anencephaly. The risk was increased, although not significantly, for fathers who were applicators when compared to agricultural workers who were not applicators.	[20]
Dichotomous pesticide exposure: None: Control: 2042 (NI); All NTDs: 334 (NI); Anencephaly: 81 (NI); Spina bifida: 210 (NI); Encephalocele: 41 (NI). Insecticides + herbicides: Control: 52 (NI); All NTDs: 19 (NI); Anencephaly: 4; Spina bifida: 14 (NI); Encephalocele: 1. Insecticides + herbicides + fungicides: Control: 211 (NI); All NTDs: 52 (NI); Anencephaly: 17 (NI); Spina bifida: 25 (NI); Encephalocele: 9 (NI). **Estimated cumulative exposure (mg):** None: Control: 2042 (NI); All NTDs: 334 (NI); Anencephaly: 81 (NI); Spina bifida: 210 (NI); Encephalocele: 41 (NI). Insecticides + herbicides: >0 and <9.482: Control: 26 (NI); All NTDs: 10 (NI); Anencephaly: 2 (NI); Spina bifida: 8 (NI); Encephalocele: 0. ≥9.482: Control: 26 (NI); All NTDs: 9 (NI); Anencephaly: 2 (NI); Spina bifida: 6 (NI); Encephalocele: 1 (NI). Pesticides + herbicides + fungicides: >0 and <245.089: Control: 105 (NI); All NTDs: 22 (NI); Anencephaly: 7 (NI); Spina bifida: 9 (NI); Encephalocele: 6 (NI). ≥245.089 Control: 106 (NI); All NTDs: 30 (NI); Anencephaly: 10 (NI); Spina bifida: 16 (NI); Encephalocele: 3 (NI).	aOR: **Dichotomous pesticide exposure:** Insecticides + herbicides: All NTDs: 1.7 (0.9–3.1); Anencephaly: NC ^c^; Spina bifida: 2.1 (1.0–4.1); Encephalocele: NC. Insecticides + herbicides + fungicides: All NTDs: 1.1 (0.8–1.6); Anencephaly: 1.6 (0.9–2.8); Spina bifida: 0.9 (0.5–1.4); Encephalocele: 9 1.6 (0.7–3.7). **Estimated cumulative exposure (mg):** Insecticides + herbicides: >0 and <9.482: All NTDs: 1.7 (0.7–3.9); Anencephaly: NC; Spina bifida: 2.1 (0.8–5.3); Encephalocele: NC. ≥9.482: All NTDs: 1.7 (0.7–4.0); Anencephaly: NC; Spina bifida: 2.0 (0.7–5.5); Encephalocele: NC. Pesticides+herbicides+fungicides: >0 and <245.089: All NTDs: 1.1 (0.6–1.7); Anencephaly: 1.4 (0.6–3.2); Spina bifida: 0.7 (0.4–1.5); Encephalocele: 2.1 (0.8–5.6). ≥245.089 Control: 106; All NTDs: 1.2 (0.8–2.0); Anencephaly: 1.8 (0.8–3.9); Spina bifida: 1.0 (0.5–1.9); Encephalocele: NC.	NI	NI	Anencephaly, craniorachischisis, spina bifida, or encephalocele	Positive associations, although marginal or non-significant, were obtained for any exposure and cumulative exposure to insecticides + herbicides + fungicides for anencephaly alone or encephalocele alone, as well as when mothers were exposed to insecticides + herbicides for all cases of NTDs combined, in which case it was significant for spina bifida alone.	[21]
NI	PRR: Mother’s work (≥500 h yearly): All neural tube defects: 0.85 (0.57–1.27) Anencephaly: 1.37 (0.70–2.70) Spina bifida: 0.78 (0.39–1.53) Hydrocephalus: 0.58 (0.28–1.21)	NI	PRR: Father’s work (≥500 h yearly): All neural tube defects: 1.62 (1.06–2.47) Anencephaly: 1.16 (0.58–2.27) Spina bifida: 1.67 (0.79–3.51) Hydrocephalus: 2.37 (1.11–5.04)	Anencephaly, spina bifida, and hydrocephaly	Potato production and paternal work on the farm for more than 500 h yearly were associated with the occurrence of NTDs. However, no influence was found for maternal occupation.	[22]
Nonexposed: Controls: 1745 (64.0) Cases (Spina bifida): 183 (64.2) Exposed: Controls: 673 (24.7) Cases (Spina bífida): 59 (20)	aOR: Any exposure: 0.8 (0.5–1.3)	Nonexposed: Controls: 1745 (64.0) Cases (Spina bifida): 183 (64.2) Exposed: Controls: 183 (6.7) Cases (Spina bífida): 18 (6.3)	aOR: Any exposure: 0.8 (0.6–1.8) Insecticide + fungicide: 1.5 (0.8–2.8)	Spina bifida	No association was found between maternal exposure and the risk of spina bifida. However, there is a slight association between combined paternal occupational exposure to insecticides and fungicides and the occurrence of spina bifida. Exposure of both parents to any type of pesticide was associated with the malformation.	[24]
Farmer: Cases: 64 (83) Controls: 45 (92) Not farmer: Cases: 13 (17) Controls: 4 (8)	NI	NI	NI	Anencephaly and spina bifida	Concentrations of o, p′-DDT, o, p′-DDE, o, p′-DDD, or the sum Σ3o, p′-DDTs), α-HCH, γ-HCH, and endosulfan were significantly higher in cases compared to those of controls. However, no statistical differences were found between mothers of cases and controls working on farms.	[25]
Farmer: Cases: 93 (80) Controls: 84 (69) Not farmer: Cases: 23 (20) Controls: 37 (31)	OR: 0.616 (0.340–1.12)	NI	NI	Anencephaly, spina bifida, and encephalocele	OCPs concentration was higher in mothers’ cases than in mothers of the controls. A slight trend of a difference in the maternal occupation of cases and controls was observed; however, no maternal occupation was found to be associated with the risk of NTD.	[28]
Farmer: Controls: 83 (70.9) Cases: 91 (78.4) Not farmer: Controls: 34 (29.1) Cases: 25 (21.6)	NI	NI	NI	Anencephaly, spina bifida, and encephalocele	Exposure to OCPs was associated with cases of NTDs. However, there was no significant difference between cases and controls with regard to occupation.	[30]

OR: Odds ratio; aOR: Adjusted odds ratio; PRR: Prevalence rate ratio. ^b^: NI: Not informed. ^c^: NC: Not calculated. ^d^: Information (n and %) of non-exposed cases and controls were not informed.

**Table 3:** Associations between geographical proximity to agricultural fields and the risk of neural tube defects.

Geographic Proximity	Culture	Cases and Control n (%)	Geographic Exposure (aOR or OR, 95% CI) ^a^	NTDs	Outcomes	Reference
100 m	They do not specify the type of crops being studied.	Cases: 15 (17.6%) Control: 7 (13%)	aOR 3.24 (0.94–12.31)	Holoprosencephaly	At 100 m, there is an association with holoprosencephaly during the preconception period or early pregnancy near agricultural fields.	[15]
<400 m	They do not specify the type of crops being studied.	Cases: Lived < 400 m: 30 (16.3%); Walked through fields: 9 (4.9%) Control: Lived < 400 m: 16 (7.1%); Walked through fields: 3 (1.3%)	Lived < 400 m: OR 3.6 (1.7–7.6); Walked through fields: OR 4.7 (1.0–30.2)	Anencephaly, spina bifida, and encephalocele	At less than 400 m, there is an association with anencephaly and spina bifida, especially for women who reported walking through fields.	[17]
No specific distance specified	They do not specify the type of crops being studied.	Cases: NTD: 7 Anencephaly: 6 Spina bifida: 1 Control: 192	NTD: OR 1.47 (0.69–3.12) Anencephaly: OR 3.06 (1.39–6.71) Spina bifida: OR 0.34 (0.05–2.21)	CNS groupings NTDs, anencephaly, and Spina bifida	No specific distance, but exposure to pesticides is associated with an increased risk of NTDs, with a stronger relationship for anencephaly.	[23]
400 m, 500 m, 800 m	Non-Permanent crops, orchard crops, and vineyards.	Any nonpermanent crops: Cases: self-reported (400 m)—64 (28.2%) Controls: self-reported (400 m)—91 (21.5%) Any orchards: Cases: self-reported (400 m)—31 (13.7%) Controls: self-reported (400 m)—36 (8.6%) Vineyards: Cases: land-use survey (400 m)–15 (6.6%) Control: land-use survey (400 m)—12 (2.9%) Cases: land-use survey (500 m)—19 (8.4%) Controls: land-use survey (500 m)—19 (4.5%)	Any nonpermanent crops: Self-reported (400 metros): OR = 1.62, IC = 1.08–2.43 Any orchards: Self-reported (400 metros): OR = 1.95, IC = 1.14–3.34 Vineyards: Land-use survey (400 metros): OR = 2.45, IC = 1.08–5.58 Land-use survey (500 metros): OR = 2.04, IC = 1.01–4.13	Anencephaly, spina bifida cystica, craniorachischis, and iniencephaly	At 400 m to 800 m, there is an association with NTDs when living near agricultural crops, with the highest risk near vineyards.	[26]
1000 m	Non-permanent crops and permanent crops.	Cases: Amides: Other NTDs: 0 (0%) Anencephaly: 12 (1.6%) Spina bifida: 12 (1.6%) Benzimidazoles: Other NTDs: 2 (0.3%) Anencephaly: 18 (2.5%) Spina bifida: 19 (2.6%) Methyl carbamates: Other NTDs: 4 (0.5%) Anencephaly: 38 (5.2%) Spina bifida: 45 (6.15%) Organophosphorus: Any NTDs: 8 (1.1%) Anencephaly: 73 (10%) Spina bifida: 69 (9.4%). Control: Amides: 15 (1.6%) Benzimidazoles: 20 (2.1%) Methyl carbamates: 77 (8.2%) Organophosphorus: 142 (15.1%).	Amides: Any NTDs: OR = 2.2 (1.0, 5.3) Anencephaly: OR = 2.1 (0.8, 5.9) Spina bifida: OR = 3.3 (1.2, 9.3) Benzimidazoles: Any NTDs: OR = 2.2 (1.1, 4.7) Anencephaly: OR = 1.8 (0.7, 4.7) Spina bifida: OR = 2.7 (1.1, 6.5) Methyl carbamates: Any NTDs: OR = 1.5 (1.0, 2.3) Anencephaly: OR = 1.3 (0.7, 2.2) Spina bifida: OR = 1.7 (1.0, 2.9) Organophosphorus: Any NTDs: OR = 1.3 (0.9, 1.8) Anencephaly: OR = 1.6 (1.0, 2.5) Spina bifida: OR = 1.1 (0.7, 1.6)	Anencephaly, spina bifida cystica, craniorachischisis, and iniencephaly.	At 1000 m, there is an association between pesticide exposure and an increased risk of NTDs, with a focus on anencephaly and spina bifida.	[27]
00 m	Infrequently rotated crops, such as orchards and vineyards, were analyzed individually, allowing for a more precise assessment. Conversely, frequently rotated Crops were grouped into a single category. Although they do not directly compare the effects, the methodology allows for some analysis of different pesticide exposures.	Spina bifida–Bromoxynil (hydroxybenzonitrile): Cases: 7 (5.69%) any exposed—116 (94%) none exposed. Control: 10 (1.3%) exposed—775 (98.7%) none exposed	Spina bifida—Bromoxynil (hydroxybenzonitrile): OR 5.1 (1.7, 15.6)	Anencephaly and spina bifida	At 500 m, there is an association between exposure to bromoxynil and an increased risk of spina bifida.	[29]

^a^: OR: Odds ratios; aOR: Adjusted odds ratios.

**Table 4:** Quality assessment tool for case control studies.

Score (Out of 12; 2 Points Were Not Applicable)	Quality (%)	Reference
7/10	Good (70%)	[15]
9/10	Good (90%)	[16]
9/10	Good (90%)	[17]
8/10	Good (80%)	[18]
9/10	Good (90%)	[19]
8/10	Good (80%)	[20]
9/10	Good (90%)	[21]
8/10	Good (80%)	[23]
9/10	Good (90%)	[24]
10/10	Good (100%)	[25]
9/10	Good (90%)	[26]
9/10	Good (90%)	[27]
9/10	Good (90%)	[28]
8/10	Good (80%)	[29]
10/10	Good (100%)	[30]

**Table 5:** Quality assessment tool for observational cohort study.

Score (Out of 14; 3 Points Were Not Applicable)	Quality (%)	Reference
9/11	Good (81.8)	[22]

## Data Availability

No new data were created or analyzed in this study. Data sharing is not applicable to this article.

## Supplementary Materials

The following supporting information can be downloaded at: https://www.mdpi.com/article/10.3390/toxics13010034/s1. Table S1: Search mechanisms performed in databases; Table S2: Quality Assessment of Case Control Studies; Table S3: Quality Assessment of Cohort Studies.
