# Supplementary material for: A Systematic Review of Parental Occupational Pesticide Exposure and Geographical Proximity to Agricultural Fields in Association with Neural Tube Defects
Source: Toxics. 2025 Jan 4;13(1):34. doi: 10.3390/toxics13010034 (PMC11769355; doi:10.3390/toxics13010034)
Supplement: Supplementary file 1 [file toxics-13-00034-s001.zip › toxics-3359642-supplementary.pdf]

**Supplementary Material**

**Table S1:** Search mechanisms performed in databases.

| Database | Search query |
|---|---|
| **Pubmed** | (("pesticidal"[All Fields] OR "pesticide s"[All Fields] OR "pesticides"[Pharmacological Action] OR "pesticides"[MeSH Terms] OR "pesticides"[All Fields] OR "pesticide"[All Fields]) AND ("occupant"[All Fields] OR "occupant s"[All Fields] OR "occupants"[All Fields] OR "occupational"[All Fields] OR "occupations"[MeSH Terms] OR "occupations"[All Fields] OR "occupation"[All Fields] OR ("exposure"[All Fields] OR "exposure s"[All Fields] OR "exposured"[All Fields] OR "exposures"[All Fields] OR "exposuring"[All Fields])) AND ("neural tube defects"[MeSH Terms] OR ("neural"[All Fields] AND "tube"[All Fields] AND "defects"[All Fields]) OR "neural tube defects"[All Fields])) |
| **ScienceDirect** | pesticides AND (occupational OR exposure) AND neural tube defects |
| **Embase** | ('pesticides'/exp OR pesticides) AND ('occupational'/exp OR occupational OR 'exposure'/exp OR exposure) AND ('neural tube defects'/exp OR 'neural tube defects' OR (neural AND ('tube'/exp OR tube) AND defects)) AND [2000-2023]/py AND ([article]/lim OR [article in press]/lim) |
| **Cochrane** | pesticides in All Text AND neural tube defects in All Text AND occupational OR exposure in All Text |
| **Web of Science** | occupational OR exposure (All Fields) and pesticides (All Fields) and neural tube defects (All Fields) and Article (Document Types) |
| **Scopus** | (ALL (occupational OR exposure) AND ALL (pesticides) AND ALL (‘neural AND tube AND defects'/exp)) AND PUBYEAR > 1999 AND PUBYEAR < 2024 AND (LIMIT-TO (DOCTYPE, "ar”)) |
| **SciELO** | pesticides AND (occupational OR exposure) AND neural tube defects |
| **BVS/LILACS** | pesticides AND (occupational OR exposure) AND neural tube defects |
| **Oxford Academic** | neural tube defects AND pesticide AND occupational |
| **ACS** | neural tube defects AND pesticide AND occupational OR paternal occupational OR parental occupational |
| **EBSCO** | neural tube defects AND pesticide AND occupational |
| **Google Scholar** | “defeitos no tubo neural” AND pesticida AND ocupacional |

**Table S2:** Quality Assessment of Case-Control Studies.

| Reference/ Question[a] | 1 | 2 | 3 | 4 | 5 | 6 | 7 | 8 | 9 | 10 | 11 | 12 | Quality |
|---|---|---|---|---|---|---|---|---|---|---|---|---|---|
| Addissie et al. (2020) | Y[b] | Y | Y | Y | Y | Y | NA[c] | NA | N[d] | N | NR[e] | Y | 70% |
| Brender et al. (2002) | Y | Y | Y | Y | Y | Y | NA | NA | Y | Y | N | Y | 90% |
| Brender et al. (2010) | Y | Y | Y | Y | Y | Y | NA | NA | Y | Y | N | Y | 90% |
| Fear et al. (2007) | Y | Y | Y | Y | Y | Y | NA | NA | Y | Y | N | N | 70% |
| Kalra et al. (2016) | Y | Y | Y | Y | Y | Y | NA | NA | Y | Y | NR | Y | 90% |
| Lacasaña et al. (2006) | Y | Y | Y | Y | Y | Y | NA | NA | N | Y | NR | Y | 80% |
| Makelarski et al. (2014) | Y | Y | Y | Y | Y | Y | NA | NA | Y | Y | NR | Y | 90% |
| Orr et al. (2022) | Y | Y | Y | Y | Y | Y | NA | NA | Y | Y | NR | NR | 80% |
| Pettigrew et al. (2016) | Y | Y | Y | Y | Y | Y | NA | NA | Y | Y | NR | Y | 90% |
| Ren et al. (2011) | Y | Y | Y | Y | Y | Y | NA | NA | Y | Y | Y | Y | 100% |
| Rull et al. (2006a) | Y | Y | Y | Y | Y | Y | NA | NA | Y | Y | N | Y | 90% |
| Rull et al. (2006b) | Y | Y | Y | Y | Y | Y | NA | NA | Y | Y | N | Y | 90% |
| Wang et al. (2014). | Y | Y | Y | Y | Y | Y | NA | NA | Y | Y | N | Y | 90% |
| Yang et al. (2014). | Y | Y | Y | Y | Y | Y | NA | NA | N | Y | NR | Y | 80% |
| Yin et al. (2021) | Y | Y | Y | Y | Y | Y | NA | NA | Y | Y | Y | Y | 100% |

[a]1. Was the research question or objective in this paper clearly stated and appropriate?
2. Was the study population clearly specified and defined?
3. Did the authors include a sample size justification?
4. Were controls selected or recruited from the same or similar population that gave rise to the cases (including the same timeframe)?
5. Were the definitions, inclusion and exclusion criteria, algorithms or processes used to identify or select cases and controls valid, reliable, and implemented consistently across all study participants?
6. Were the cases clearly defined and differentiated from controls?

7. If less than 100 percent of eligible cases and/or controls were selected for the study, were the cases and/or controls randomly selected from those eligible?
8. Was there use of concurrent controls?
9. Were the investigators able to confirm that the exposure/risk occurred prior to the development of the condition or event that defined a participant as a case?
10. Were the measures of exposure/risk clearly defined, valid, reliable, and implemented consistently (including the same time period) across all study participants?
11. Were the assessors of exposure/risk blinded to the case or control status of participants?
12. Were key potential confounding variables measured and adjusted statistically in the analyses? If matching was used, did the investigators account for matching during study analysis?
[b]Y: yes;
[c]NA: not applicable;
[d]N: no;
[e]NR: not reported

**Table S3:** Quality Assessment of Observational Cohort Study.

| Reference/ Question[a] | 1 | 2 | 3 | 4 | 5 | 6 | 7 | 8 | 9 | 10 | 11 | 12 | 13 | 14 | Quality |
|---|---|---|---|---|---|---|---|---|---|---|---|---|---|---|---|
| Nordby et al. (2005) | Y[b] | Y | Y | Y | Y | NA[c] | Y | Y | Y | NA | Y | N[d] | NA | N | 81.8% |

[a]1. Was the research question or objective in this paper clearly stated?
2. Was the study population clearly specified and defined?
3. Was the participation rate of eligible persons at least 50%?
4. Were all the subjects selected or recruited from the same or similar populations (including the same time period)? Were inclusion and exclusion criteria for being in the study prespecified and applied uniformly to all participants?
5. Was a sample size justification, power description, or variance and effect estimates provided?;
6. For the analyses in this paper, were the exposure(s) of interest measured prior to the outcome(s) being measured?
7. Was the timeframe sufficient so that one could reasonably expect to see an association between exposure and outcome if it existed?
8. For exposures that can vary in amount or level, did the study examine different levels of the exposure as related to the outcome (e.g., categories of exposure, or exposure measured as continuous variable)?
9. Were the exposure measures (independent variables) clearly defined, valid, reliable, and implemented consistently across all study participants?
10. Was the exposure(s) assessed more than once over time?
11. Were the outcome measures (dependent variables) clearly defined, valid, reliable, and implemented consistently across all study participants?
12. Were the outcome assessors blinded to the exposure status of participants?
13. Was loss to follow-up after baseline 20% or less?
14. Were key potential confounding variables measured and adjusted statistically for their impact on the relationship between exposure(s) and outcome(s)?
[b]Y: yes;
[c]NA: not applicable;
[d]N: no;
